# Language Comprehension Developmental Milestones in Typically Developing Children Assessed by the New Language Phenotype Assessment (LPA)

**DOI:** 10.3390/children12060793

**Published:** 2025-06-17

**Authors:** Andrey Vyshedskiy, Ariella Pevzner, Brigid Mack, Eva Shrayer, Miranda Zea, Sasha Bunner, Nichole Wong, Elena Baskina, Amira Sheikh, Alessandro Tagliavia, Andriane Schmiedel Fucks, Andressa Schmiedel Sanches Santos, Lucas Ernesto Pavoski Poloni, Elielton Fucks, Yudit Bolotovsky, Sung Jin (Sam) Kang

**Affiliations:** 1 Boston University, Boston, MA 02215, USA; 2 New York University, New York, NY 10012, USA; 3 Independent Researcher, Boston, MA 02135, USA; 4 Soulmare—Autism Clinic, Foz do Iguaçu 85851-240, Brazil

**Keywords:** autism, language therapy, developmental psychology, theory of mind, language assessment, early intervention, language comprehension

## Abstract

**Background/Objectives**: Three distinct language comprehension phenotypes have previously been identified in individuals with language deficits: (1) individuals with the Command Phenotype are limited to understanding simple commands; (2) individuals with the Modifier Phenotype demonstrate additional comprehension of combinations of nouns and adjectives; and (3) individuals with the Syntactic Phenotype possess full syntactic comprehension. We hypothesized that typically developing children progress through these same three language comprehension phenotypes and aimed to determine the typical age at which each phenotype emerges. **Methods**: To assess comprehension in young children, we developed the 15-item *Language Phenotype Assessment* (LPA). This tool uses toy-animal manipulatives to avoid reliance on picture interpretation and employs brief instructions to reduce auditory memory load. LPA items incorporate elements such as colors, sizes, numbers, spatial prepositions, and other syntactic components, posing novel combinations of words that children had not previously encountered. The LPA was administered to 116 typically-developing children aged 1.5–7 years, recruited by approaching parents in local parks and inviting them to participate. **Results**: Findings revealed a developmental trajectory consistent with the three previously described phenotypes: 50% of children attained the Command Phenotype by 1.6 years of age, the Modifier Phenotype by 3.0 years of age, and the Syntactic Phenotype by 3.7 years of age. All children acquired the Command Phenotype by 3, the Modifier Phenotype by 4, and the Syntactic Phenotype by 5 years of age. **Conclusions**: The LPA is an effective tool for assessing comprehension in children aged 1.5–5 years. It allows for the early identification of comprehension difficulties, supporting the timely initiation of appropriate language interventions.

## 1. Introduction

Language comprehension development is a complex process marked by distinct milestones at various stages of early childhood [1,2]. In infancy, children begin discerning subtle nuances in speech by distinguishing phonetic sounds. As they enter toddlerhood, comprehension expands to include vocabulary growth and understanding of grammatical structures. Through interactions with caregivers and exposure to a range of linguistic stimuli, children refine their comprehension skills, learning to interpret contextual cues and infer meaning from conversations and narratives. This developmental journey is driven by a dynamic interplay between genetic predispositions and nurture [3,4,5]. Both suboptimal genes and language deprivation can hinder language acquisition [6]. Deleterious genetic variations, for example, are the leading cause of Autism Spectrum Disorder (ASD), and as many as 40% of individuals diagnosed with ASD do not attain the Syntactic Language Comprehension Phenotype [7]. At the same time, genetically-typical individuals who were not engaged in syntactic conversations in early childhood also exhibit lifelong deficits in syntactic language comprehension [6,8,9,10,11,12,13,14,15,16]. Early language therapy interventions can often mitigate the impact of suboptimal genes and language deprivation [17,18,19,20,21,22]. Consequently, there is a strong interest in the timely identification of language deficits in young children to ensure early and effective intervention [23,24,25,26].

Recent studies have identified three distinct language comprehension phenotypes in autistic individuals, offering a valuable opportunity to enhance the detection and characterization of language deficits in young children [27]. Analysis of language comprehension across varying levels of complexity in over 31,000 autistic individuals revealed three distinct phenotypes that remained stable across age groups: (1) individuals with the Command Phenotype were limited to comprehension of single words and simple commands; (2) individuals with the Modifier Phenotype demonstrated additional ability to understand sentences combining nouns with adjectives such as color, size, and number; and (3) individuals with the Syntactic Phenotype added comprehension of spatial prepositions, verb tenses, flexible syntax, possessive pronouns, complex explanations, and fairy tales.

The existence of the three distinct language phenotypes was later confirmed in an expanded pool of participants that included other conditions linked to language impairments: mild language delay, apraxia (a motor speech disorder where individuals struggle to plan and coordinate the movements needed for speech despite normal muscle function), Specific Language Impairment, Sensory Processing Disorder, Social Communication Disorder, Down Syndrome, and Attention-Deficit/Hyperactivity Disorder (ADHD) [28].

However, the acquisition of language phenotypes by typically developing children has not been explored. We hypothesized that typically developing children progress through these same stages of language comprehension observed in populations with language deficits: the Command, Modifier, and Syntactic Phenotypes. If confirmed, establishing normative data for the acquisition of these language comprehension phenotypes could serve as a valuable tool for the early identification and monitoring of language development in children. Such norms would provide a benchmark for distinguishing typical from atypical language comprehension patterns, ultimately supporting earlier diagnosis and more targeted interventions.

Accordingly, we reviewed all available assessments that could potentially characterize an individual’s language comprehension phenotype. The ideal instrument would be capable of assessing a range of linguistic abilities with gradually increasing complexity: from the Command level to the Modifier level, and ultimately to the Syntactic level [27].

Previous research suggested that typically developing children acquire the Command Phenotype by 2 years and the Syntactic Phenotype by 4 years of age [29,30]. Hence, we focused our search on language comprehension assessments for children aged 2 to 4. To our surprise, most available tools for early language learners, such as the Peabody Picture Vocabulary Test (PPVT-4) [31] and the Expressive Vocabulary Test (EVT-2) [32], do not evaluate word-combination skill. Instead, they focus exclusively on measuring vocabulary. This approach fails to directly evaluate a language comprehension phenotype, potentially leading to inaccurate language assessments, since atypical individuals with any language comprehension phenotype can learn an unlimited number of words [10,15].

Four language comprehension assessments were identified that focused on evaluating language comprehension at the sentence-level: the Preschool Language Scales (PLS-5) [33], the Token Test for children [34,35], the Clinical Evaluation of Language Fundamentals (CELF-5) [36], and the Test for Reception Of Grammar (TROG) [37]. All these assessments, however, had significant drawbacks (Table 1).

The PLS-5 has been developed and validated for use with children from birth to 8 years of age. However, most items targeted at younger children are based on word-level comprehension. Sentence-level comprehension is assessed by overly complex sentences (see Note 1 in Table 1). As a result, PLS-5 is not suited for evaluating language comprehension phenotypes in young children in the age range between 2 and 4 years.

The Token Test is designed for children 3 to 14 years of age. However, it also suffers from overly complex instructions (see Note 2 in Table 1). The longer-than-necessary instructions of the Token Test make it suboptimal for the assessment of language comprehension phenotypes in young language learners in the age range between 2 and 4 years.

The Clinical Evaluation of Language Fundamentals (CELF-5) [36] is standardized for students 5 to 21 years of age, and the Test for Reception of Grammar (TROG) [37] is standardized for students 4 to 18+ years of age, making them unsuitable for assessment of language comprehension phenotypes in children 2 and 4 years of age. Both CELF-5 and TROG have an additional problem of requiring participants to answer by pointing to a correct picture, rather than showing the answer with tangible objects (manipulatives). Children with ASD and attention deficit disorder often fail to interpret picture answers [38].

Thus, none of the existing language comprehension tests were suitable for assessing progression over the Command, Modifier, and Syntactic Phenotypes acquired between 2 and 4 years of age. Accordingly, it was necessary to develop a new assessment that could evaluate the trajectory of language comprehension toward the Syntactic Phenotype. In the future, such a tool could enable tracking syntactic language acquisition in individuals with impairments, thereby facilitating a timely language therapy intervention. The resulting 15-item Language Phenotype Assessment (LPA) uses toy-animal manipulatives and brief language instructions to minimize auditory memory demands. The LPA was tested in a convenience sample of 116 typically developing children.

## 2. Methods

The Language Phenotype Assessment (LPA) is rooted in a set of common language comprehension items whereby the participants are required to follow verbal commands of increasing difficulty. The LPA consists of 15 items: three items at the Command language levels, five items at the Modifier language level, and seven items at the Syntactic level (Table 2). All items are scored as either *1*: participant demonstrated an understanding of the item, or *0*: participant did not demonstrate an understanding of the item. The LPA total score was calculated based on the number of items completed correctly. A total score of 15 indicates that a participant demonstrated an understanding of all items. Similarly, a participant who demonstrated an understanding of seven items would receive a total score of 7; a participant who demonstrated an understanding of no items would receive a total score of 0, and so on.

The entire test is designed to take approximately 10 min to complete. Test items are administered in a fixed order. Prior to the evaluation, the names of manipulatives, colors, sizes, and spatial prepositions are demonstrated to the child. A detailed description of each item is provided in Table 2. Each item consists of four tasks. To demonstrate their understanding of an item, a child must correctly complete at least three out of the four tasks. The complete LPA form can be found in the Supplementary Material.

### 2.1. Manipulatives

The LPA uses the following manipulatives (Figure S1): (1) Large (18 cm) pencils of 4 colors: red, blue, green, and yellow. (2) Small pencils (1/3 length of large pencils) of 4 colors: red, blue, green, and yellow. (3) Large (25 cm) straws of 4 colors: red, blue, green, and yellow (three of each color). (4) Small straws (1/3 length of large straws) of 4 colors: red, blue, green, and yellow (one of each color). (5) A set of puppet-like plush animals (20 cm × 7 cm × 5 cm): giraffe, lion, elephant, and monkey. 6) A set of four colored cups (10 cm × 7 cm × 7 cm): red, blue, green, and yellow.

**1.**  **Command item—give me an animal**

For this item, puppet-like plush animals (giraffe, lion, elephant, and monkey, Figure S1) were placed on a flat surface. Each participant was asked to identify the animals to confirm basic knowledge of animal names. If they did not know the name, the name was repeated three times. At least 75% accuracy was required to earn a score of *1*. This 75% accuracy threshold was chosen to accommodate possible lapses in attention. With four animals, the probability of answering 75% of tasks correctly by chance is 4.7%.

**2.**  **Command item—give a cup or a pencil to an animal**

Again, the giraffe, lion, elephant, and monkey were laid on a flat surface, each placed as far apart from the others as possible. A cup and a pencil were positioned nearby. Participants were asked to give a cup or a pencil to an animal. After each task, the tester encouraged the child by saying “Good job”, but no feedback was given concerning the correctness of the answer to prevent the child from memorizing the answers. At least 75% accuracy was required to earn a score of *1*. The probability of answering 75% of tasks correctly by chance is 0.68%

**3.**  **Command item—take an animal to another animal**

As before, the giraffe, lion, elephant, and monkey were laid on a flat surface, each placed as far apart from the others as possible. Each participant was asked to bring one animal to another animal. At least 75% accuracy was required to earn a score of *1*. With four animals, the probability of answering 75% of tasks correctly by chance is 1.5%.

**4.**  **Modifier item—color integration**

Integration of color requires the participants to integrate a noun and an adjective. Participants were asked to select an object (e.g., *red* straw) placed among 24 objects (4 large pencils of different colors, 4 small pencils of different colors, 12 large straws of different colors, 4 small straws of different colors), thus forcing the participant to notice and integrate both color and object. Prior to completing this item, participants were asked to point to and name the color of straws and pencils to confirm that they understood the word for specific colors. Participants needed to answer correctly at least 3 out of 4 tasks (75% accuracy) to receive a score of *1* for this item. The probability of answering 75% of tasks correctly by chance is <1.5%.

**5.**  **Modifier item—size integration**

Integration of size requires the participants to integrate a noun and an adjective. Participants were asked to select an object (e.g., *big* straw) placed among 24 objects listed in item 4, thus forcing the participant to notice and integrate size and object. Prior to completing this item, participants were asked to point to and name the size of various objects to confirm that they understood the words *big* and *small*. Participants needed to answer correctly at least 3 out of 4 tasks (75% accuracy) to receive a score of *1* for this item. The probability of answering 75% of tasks correctly by chance is <1.5%.

**6.**  **Modifier item—color and size integration**

Participants were asked to select an object (e.g., *long red* straw) placed among 24 objects listed in item 4, thus forcing the participant to notice and integrate color, size, and object. Participants needed to answer correctly at least 3 out of 4 tasks (75% accuracy) to receive a score of *1* for this item. The probability of answering 75% of tasks correctly by chance is 0.68%.

**7.**  **Modifier item—number integration**

Participants were asked to select two or three objects (e.g., *three* straws) placed among 24 objects listed in item 4. Participants needed to answer correctly at least 3 out of 4 tasks (75% accuracy) to receive a score of *1* for this item. The probability of answering 75% of tasks correctly by chance is <1%.

**8.**  **Modifier item—number and color integration**

Participants were asked to select two or three objects of a specific color (e.g., *three red* straws) placed among 24 objects listed in item 4. Participants needed to answer correctly at least 3 out of 4 tasks (75% accuracy) to receive a score of *1* for this item. The probability of answering 75% of tasks correctly by chance is <1%.

**9.**  **Syntactic item—stacking cups, canonical word order**

A set of four colored cups (Figure S1) was used for this test. The purpose of this task was to determine whether participants could properly arrange two cups, based on verbal instructions. Before the test, participants were given a demonstration of how to “put the *blue* cup inside the *red* cup” and, if necessary, were helped to stack the cups correctly. This training session with the *blue* and *red* cups was repeated while randomly switching the cup order until the participant was able to stack the correct cups on their own with no errors. Once participants were comfortable stacking the two training cups, they were asked to stack four cups of various color combinations (Table 2, ‘Tasks’ column). Once the cups were stacked, each task was recorded as correct or incorrect. Participants needed to answer correctly at least 3 out of 4 tasks (75% accuracy) to receive a score of *1* for this item. With four cup colors, the probability of answering 75% of tasks correctly by chance is 0.2%.

**10.**  **Syntactic item—stacking cups, noncanonical word order**

The directions for stacking cups were varied syntactically from the previous item. For example, participants were instructed: “inside the blue cup, put the green cup” or “inside the red cup, put the green cup.” This was intended to be more difficult than a canonical instruction, such as “put the green cup inside the blue cup.” Participants needed to answer correctly at least 3 out of 4 tasks (75% accuracy) to receive a score of *1* for this item. With four cup colors, the probability of answering 75% of tasks correctly by chance is 0.2%.

**11.**  **Syntactic item—spatial prepositions, canonical word order**

In this item, participants were instructed to maneuver the plush animals according to the spatial prepositions *on top of* and *under*. Before the test, participants were given a demonstration of how to “put the monkey *on top of* and *under* the lion.” This training session with the monkey and lion was repeated while randomly switching the order of animals until the participant was able to stack the animals on their own with no errors. Once subjects were comfortable stacking the two training animals, participants were asked to show “the giraffe under the monkey” or “the elephant on top of the giraffe.” The pair containing the monkey and lion was not used in the actual test. The spatial prepositions *behind* and *in front of* were not used to avoid confusion about whether the perspective was from the experimenter or the participant. Participants needed to answer correctly at least 3 out of 4 tasks (75% accuracy) to receive a score of 1 for this item. With four animals, the probability of answering 75% of tasks correctly by chance is 0.2%.

**12.**  **Syntactic item—spatial prepositions, noncanonical word order**

The directions for spatial prepositions were varied syntactically from the previous item. For example, participants were instructed: “under the monkey, put the giraffe.” This was intended to be more difficult than a canonical instruction, such as “put the giraffe under the monkey.” Identically to all other items, at least 75% accuracy was required to earn a score of *1*. With four animals, the probability of answering 75% of tasks correctly by chance is 0.2%.

**13.**  **Syntactic item—spatial prepositions next to, behind, in front, between**

Participants were instructed in the following way: “Put the monkey next to you,” “Put the lion in front of you,” “Put the elephant behind you,” and “Put the giraffe between you and I.” Identically to all other items, at least 75% accuracy was required to earn a score of *1*. With four animals, the probability of answering 75% of tasks correctly by chance is <0.1%.

**14.**  **Syntactic item—mental reasoning with an object and a subject**

In the final two items, participants were asked to synthesize multiple pieces of information to solve simple mental reasoning tasks. For example, the prompt could be: “If a boy washed a girl, who is clean?” or “If a tiger ate a lion, who has a full belly”? The four tasks are mixed in such a manner that always picking the first object or always picking the second object would result in two correct responses. Identically to all other items, at least 75% accuracy was required to earn a score of *1*. The probability of answering 75% of tasks correctly by chance is 25%.

**15.**  **Syntactic item—mental reasoning with an object and a subject, passive voice**

In the final item, participants were asked to synthesize multiple pieces of information to solve simple mental reasoning tasks given in passive voice. No tangible objects were used as representations. For example, the prompt could be: “if the boy showered the girl, who is wet?” or “if a tiger was eaten by a lion, who has a full belly”? The four tasks are mixed in such a manner that always picking the first object or always picking the second object would result in two correct responses. Identically to all other items, at least 75% accuracy was required to earn a score of *1*. The probability of answering 75% of tasks correctly by chance is 25%.

### 2.2. Parent-Reported Language Phenotype Evaluation Checklist (LPEC)

While children were evaluated with LPA, we asked parents to evaluate their children’s language comprehension abilities using the *Language Phenotype Evaluation Checklist* (LPEC), shown in Table 3. To enhance compatibility, the LPEC and LPA have the same structure: three Command-level items, five Modifier-level items, and seven Syntactic-level items. The Modifier- and Syntactic-level items are identical to those found in the validated parent-reported language comprehension tool, *Mental Synthesis Evaluation Checklist* (MSEC) [30,39,40]. The Command-level items (1 to 3) are newly introduced. Response options were: ‘very true’ (2 points), ‘somewhat true’ (1 point), and ‘not true’ (0 points). A higher score indicates better language comprehension abilities.

### 2.3. Neurotypical Participants

Participants for this study were recruited by approaching parents of young children in local parks and inviting them to allow a researcher to administer a language test to their child. Children with parent-reported conditions known to impact language were excluded from the study. The data presented in this manuscript include everyone who agreed to be tested, except two children whose parents indicated that they had a developmental delay. Although no data were collected on socioeconomic status or language exposure, the location of the parks in affluent areas suggests that participants likely had high socioeconomic status and optimal language exposure. All participants’ caregivers consented to anonymized data analysis and publication of the results. All assessments were completed in a single session. The testing environment was not controlled (local parks). The final sample consisted of 116 neurotypical participants, with a mean age of 4.34 ± 1.4 years (range: 1.5–7 years), of whom 46% were male.

### 2.4. Participants Diagnosed with ASD

Participants with a diagnosis of ASD were attending Soulmare—Autism Clinic, Foz do Iguaçu, Brazil. Participants were recruited by contacting their parents. All children whose parents agreed to participate in the study were included in the study (N = 79). After obtaining informed consent from a parent, each child was evaluated by a trained clinician. All assessments were completed in a single session. The testing environment was controlled (a quiet room). The participant age range was 2 to 8 years (mean 5.0 ± 1.2 years); 72% were males.

### 2.5. Statistical Analysis

The LPA total score over age and the corresponding percentile curves were generated using Quantile Generalized Additive Models (Quantile GAMs). While traditional linear models assume a constant relationship between variables, Quantile GAMs allow for non-linear modeling that adapts to the data’s structure. This is particularly helpful when modeling developmental data like language acquisition, where growth trajectories often follow non-linear patterns [41]. By directly estimating specific percentiles (e.g., 5th, 10th, 50th), Quantile GAMs offer insights into how different points in the distribution can shift with age. This approach allowed us to represent growth curves at multiple levels of the distribution, providing a more comprehensive understanding of developmental variation across individuals [42]. Additionally, by fitting separate curves for each percentile, Quantile GAMs account for skewed distributions and changes in variance over time, both of which are common in developmental data [43]. Therefore, Quantile GAMs provide a reliable and versatile approach for mapping out age-specific growth patterns without imposing restrictive assumptions, making them highly suitable for percentile-based growth analyses.

Age-related acquisition of each language phenotype and each item was modeled using the sigmoidal function: *100/(1 + e((t_c_ − t)/τ)*, where *t_c_* represents the midpoint of the curve (50%) and *τ* controls the slope. This model was optimized using the R (version 2023.03.0) function ‘nlsLM’ (Non-linear Least-Squares) from the minpack.lm package [44].

The study adhered to all local regulations. Using the Department of Health and Human Services regulations found at 45 CFR 46.101(b)(4), the Biomedical Research Alliance of New York LLC Institutional Review Board (IRB) determined that this research project is exempt from IRB oversight (Protocol Pro00024777, BRANY Stamp Version Date: 29 March 2022). In Brazil, where participants with ASD were enrolled, the study received IRB approval from Centro Universitário Dinâmica das Cataratas (Foz do Iguaçu, Brazil, Number 8527; Date: 8 February 2021).

## 3. Results

### 3.1. Clinician-Observed Language Phenotype Assessment (LPA)

**Figure 1:**
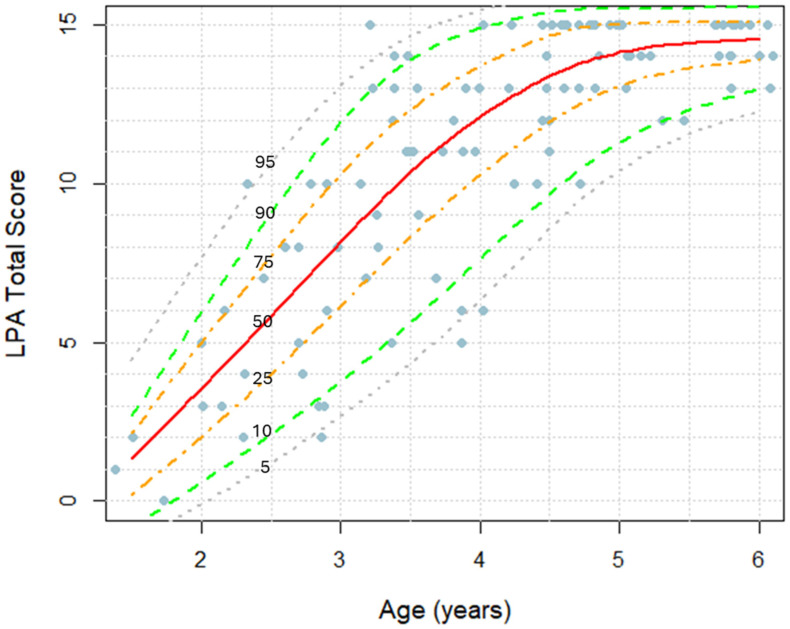
Clinician-observed LPA total score as a function of age in neurotypical children. Markers represent LPA scores in individual children. Percentile lines at 5%, 10%, 25%, 50%, 75%, 90%, and 95% are indicated.

A total of 116 neurotypical participants completed the *Language Phenotype Assessment* (LPA), Table 2. Each participant was asked to complete 15 items, with each item consisting of four distinct tasks. An item was scored as ‘1’ if at least three out of four tasks were completed correctly, indicating the participant’s understanding; otherwise, it was scored as ‘0’. Figure 1 illustrates the total LPA score as a function of age, with markers representing individual children’s scores. The near-linear increase in LPA scores between the ages of 1.5 and 5 years is followed by a plateau caused by the ceiling effect. Females scored slightly higher than males, although this difference was not statistically significant (Figure S2, Mann–Whitney U test: *p* = 0.58).

**Figure 2:**
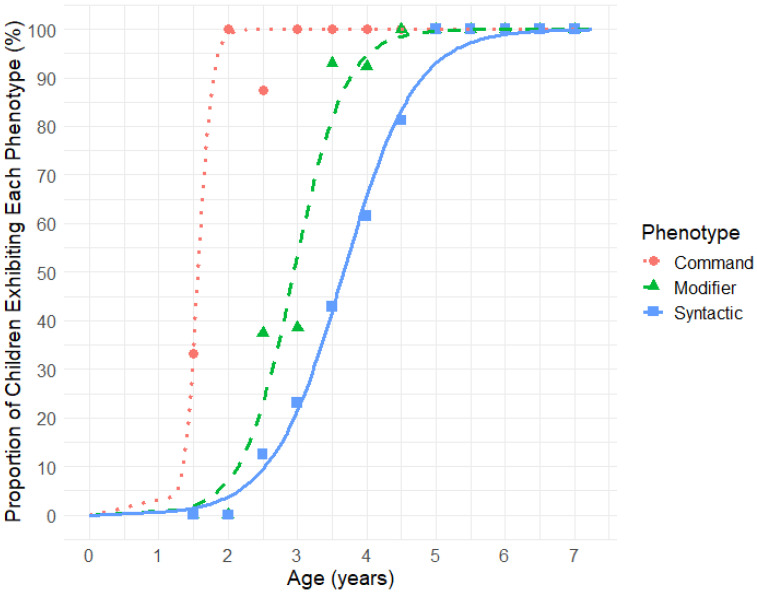
Age-related acquisition of the Command, Modifier, and Syntactic comprehension phenotypes based on clinician-observed Language Phenotype Assessment (LPA) in neurotypical children. Markers represent the proportion of children exhibiting each phenotype, calculated in 0.5-year bins: from 1.25 to 1.75 years, 1.75 to 2.25 years, and so on.

To determine the acquisition age for the Command, Modifier, and Syntactic Phenotypes, we applied the following criteria: (1) the Command Phenotype was assigned if a participant demonstrated understanding of at least two out of three Command items (items 1 to 3, Table 2); (2) the Modifier Phenotype was assigned if a participant demonstrated understanding of at least three out of five Modifier items (items 4 to 8); and (3) the Syntactic Phenotype was assigned if a participant demonstrated understanding of at least four out of seven Syntactic items (items 9 to 15). Figure 2 displays sigmoidal curves illustrating the percentage of children acquiring each phenotype as a function of age: 50% of children attained the Command Phenotype by 1.6 years, the Modifier Phenotype by 3.0 years, and the Syntactic Phenotype by 3.7 years of age. All children acquired the Command Phenotype by 3 years, the Modifier Phenotype by 4 years, and the Syntactic Phenotype by 5 years of age.

Figures S3–S17 present sigmoidal curves depicting the percentage of children acquiring each LPA item as a function of age. Table S1 provides a summary of the median age of attainment for each item, offering a concise overview of the developmental milestones median. The results show a clear developmental progression in language comprehension, with simpler Command-level items understood between 1.4 and 1.8 years, Modifier-level items between 2.3 and 3.4 years, and more complex Syntactic-level items between 2.9 and 4.9 years.

### 3.2. Psychometric Characteristics of LPA

Internal consistency was excellent (Cronbach’s alpha equals 0.92), suggesting high reliability. The item-total correlations for items 4 to 15 ranged from 0.54 to 0.79, indicating that these items contribute meaningfully to the overall scale. The item-total correlations for items 1 to 3 ranged from 0.24 to 0.45, which is expected since the majority of participants had already attained an understanding of these items at the time of the assessment. The LPA test-retest reliability was evaluated by calculating a Pearson’s Correlation between the first administration of the LPA and the re-administration of the LPA to the same participants approximately 4 months (average: 111 ± 59 days, range: 29–234 days) later (20 participants). The 4 month test-retest correlation coefficient for LPA was *r* = 0.96 (*p* < 0.0001), revealing excellent LPA long-term stability. The inter-observer agreement was very high as indicated by Kappa analysis with Kappa = 0.97 and the percent overall agreement = 98.7%.

### 3.3. Parent-Reported Language Phenotype Evaluation Checklist (LPEC)

**Figure 3:**
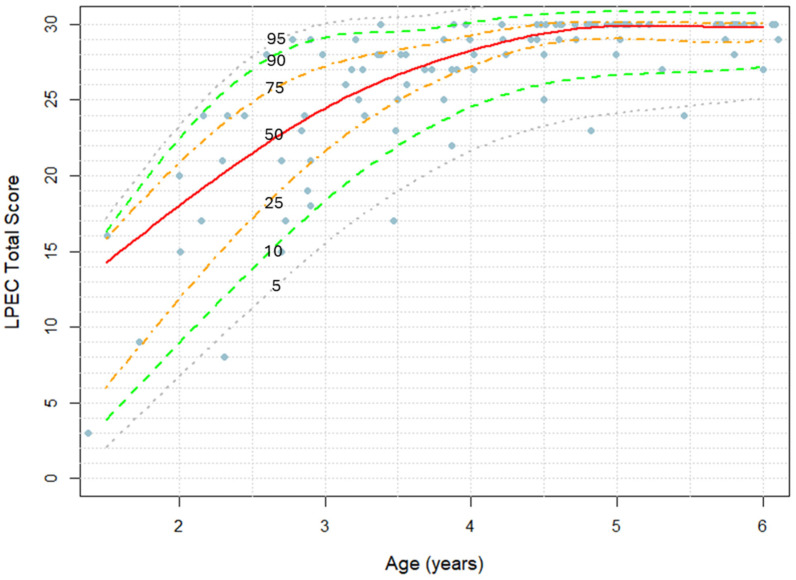
Parent-reported LPEC total score as a function of age in neurotypical children. Markers represent LPEC scores in individual children. Percentile lines at 5%, 10%, 25%, 50%, 75%, 90%, and 95% are indicated.

In addition to the clinician-administered LPA, we asked parents to evaluate their children’s language comprehension abilities using the *Language Phenotype Evaluation Checklist* (LPEC), shown in Table 3. To enhance compatibility, the LPEC and LPA share a similar structure: three Command-level items, five Modifier-level items, and seven Syntactic-level items. Figure 3 illustrates the total LPEC score by age, with markers representing individual children’s scores. A near-linear increase in LPEC scores between ages 1.5 and 5 is followed by a plateau caused by the ceiling effect. Females scored slightly higher than males, although this difference was not statistically significant (Figure S18, Mann–Whitney U test: *p* = 0.60).

**Figure 4:**
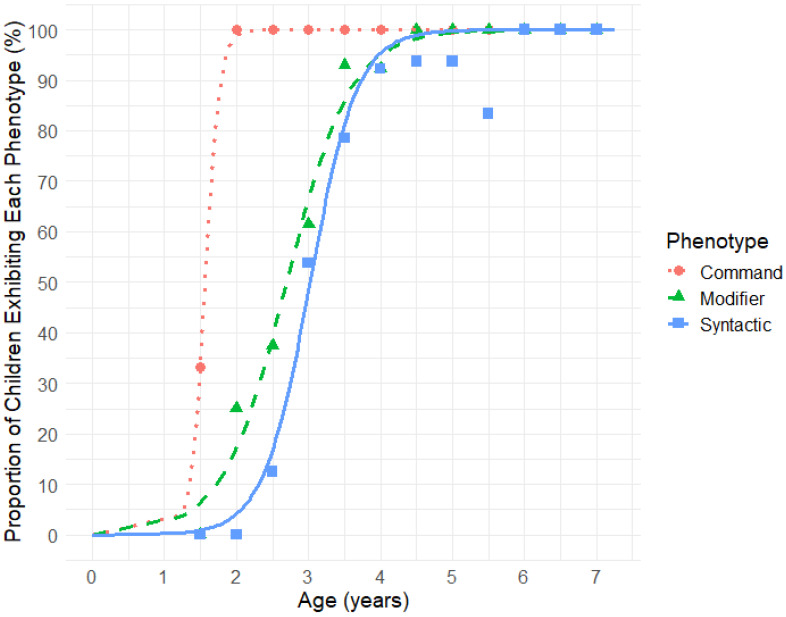
Age-related acquisition of the Command, Modifier, and Syntactic comprehension phenotypes based on parent-reported Language Phenotype Evaluation Checklist (LPEC) neurotypical children. Markers represent the proportion of children exhibiting each phenotype, calculated in 0.5-year bins.

To identify the age at which parents perceive their children as having acquired the Command, Modifier, and Syntactic Phenotypes, we applied the same criteria used in the clinician-administered LPA assessment: (1) the Command Phenotype was assigned if a child demonstrated understanding of at least two out of three command items (items 1 to 3, Table 3); (2) the Modifier Phenotype was assigned if a child demonstrated understanding of at least three out of five modifier items (items 4 to 8); and (3) the Syntactic Phenotype was assigned if a child demonstrated understanding of at least four out of seven syntactic items (items 9 to 15). Figure 4 displays sigmoidal curves showing the percentage of children attaining each phenotype. Median acquisition ages reported by parents were 1.6 years for the Command Phenotype, 2.7 years for the Modifier Phenotype, and 3.0 years for the Syntactic Phenotype. According to parent reports, all children acquired the Command Phenotype by age 3, the Modifier Phenotype by age 4, and all but one child achieved the Syntactic Phenotype by age 5.

**Figure 5:**
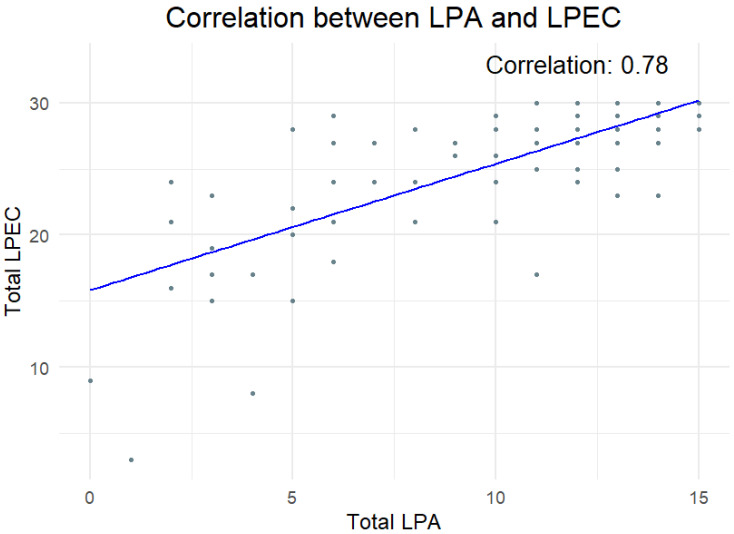
Correlation between clinician-observed LPA score and parent-reported LPEC score in neurotypical children. Each marker represents an individual child score.

To evaluate the relationship between clinician-reported LPA scores and parent-reported LPEC scores, Pearson’s correlation analysis was performed. A strong positive correlation was observed (*r* = 0.78, *p* < 0.0001), which demonstrates a significant alignment between the two assessment methods (Figure 5). Additionally, we assessed the level of agreement between clinician-assigned and parent-reported language phenotypes (Command, Modifier, and Syntactic) using Cohen’s weighted kappa. The analysis revealed substantial agreement, with a weighted kappa = 0.73, 95% CI [0.60, 0.86].

Figures S19–S33 illustrate sigmoidal curves showing the percentage of children acquiring each LPA item as a function of age. Table S2 summarizes the median age at which each item is acquired according to parents. The results show a clear developmental progression in language comprehension, with simpler Command-level items understood between 1.4 to 1.7 years, Modifier-level items between 1.9 and 3.4 years, and more complex Syntactic-level items between 2.6 and 3.9 years.

In addition to the 15-item LPEC, parents provided answers to nine items listed in Table S3. These items represent important developmental milestones indirectly related to syntactic language acquisition, such as representational drawing abilities (Figures S34 and S35), engagement in pretend play (Figure S36), and arithmetic abilities (Figures S37–S42) [45]. Table S4 summarizes the median age at which each item is acquired according to parents. Additionally, Figure S43 shows normative data for the LPEC extended with items listed in Table S3. Finally, Figure S44 presents normative data for the previously developed Mental Synthesis Evaluation Checklist (MSEC) [30,39,40], derived from LPEC results (Table S5).

### 3.4. Psychometric Characteristics of Parent-Reported LPEC

Internal consistency was excellent (Cronbach’s alpha equals to 0.91), suggesting high reliability. The item-total correlations for items 4 to 15 ranged from 0.57 to 0.79, indicating that these items contribute meaningfully to the overall scale. The item-total correlations for items 1 to 3 ranged from 0.29 to 0.50, which is expected since the majority of participants had already attained an understanding of these items at the time of the assessment. The LPEC test-retest reliability was evaluated by calculating a Pearson’s Correlation between the first administration of the LPEC and the re-administration of the LPEC to the same participants approximately 4 months (average: 111 ± 59 days, range: 29–234 days) later (20 participants). The 4-month test-retest correlation coefficient for LPEC was *r* = 0.85 (*p* < 0.0001), revealing excellent LPEC long-term stability.

### 3.5. LPA Generalizability to Children Diagnosed with Autism Spectrum Disorder (ASD)

One of key goals of the LPA is to monitor and optimize language therapy for children diagnosed with ASD. Therefore, it was critical to establish the generalizability of LPA for this population. For this purpose, we administered LPA to 79 children 2 to 8 years of age (mean 5.0 ± 1.2 years) diagnosed with ASD. While all neurotypical children 5 years of age or older attained the Syntactic Phenotype, only 39% of participants diagnosed with ASD reached this milestone (Figure S45). When assessed using the parent-reported LPEC, all but one neurotypical children 5 years of age or older attained the Syntactic Phenotype, compared to only 45% of their peers diagnosed with ASD (Figure S46).

## 4. Discussion

The importance of early language development is well-established [46,47,48,49]. Numerous laws and regulations aim to identify vulnerable individuals, such as those with congenital deafness and ASD, and to provide language therapy as soon as possible. In 1999, the U.S. Congress enacted the “Newborn and Infant Hearing Screening and Intervention Act,” which provides grants to help states establish hearing screening programs for newborns. Otoacoustic Emissions Testing is typically performed at birth, followed by an Auditory Brainstem Response assessment if the initial results suggest potential hearing loss. These screenings enable parents to introduce formal sign language to deaf children soon after birth, thus preventing delays in their exposure to syntactic language. Congenitally deaf children who are exposed to formal sign language early typically exhibit no impairment in their syntactic language comprehension [50]. This highlights the pressing need to expand these programs to support more children with language comprehension difficulties. A significant challenge in this endeavor is the early identification of vulnerable children.

It is widely recognized that children’s development follows a predetermined time course with a predictable sequence of developmental stages [51]. However, milestones for language comprehension have not been defined with the granularity required for clinical assessment of young children. This study represents a crucial first step toward establishing developmental norms for syntactic language comprehension. Once these norms have been established, assessing children against these benchmarks could become a part of a standard approach for the early identification of language deficits. Early diagnosis can, in turn, facilitate timely intervention therapy, significantly enhancing outcomes for children at risk.

An additional benefit of this assessment tool is its potential to improve the classification of language comprehension abilities. Currently, individuals’ communication levels are often categorized solely based on their verbal abilities—as *nonverbal*, *minimally-verbal*, or *verbal*. This one-dimensional framework is insufficient for accurately characterizing communication abilities. For example, a nonverbal individual with a Syntactic Phenotype may possess normal communication skills, albeit in a nonverbal format. In contrast, a verbal individual with only a Command Phenotype lacks effective communication abilities across any modality. A two-dimensional classification that incorporates both verbal abilities and language comprehension levels provides a more nuanced and informative description of an individual’s communication skills. The identification of three distinct language comprehension phenotypes—Command, Modifier, and Syntactic [27,28]—presents an opportunity to refine the characterization and monitoring of language comprehension abilities. However, this refinement necessitates a sensitive and robust assessment instrument.

The conventional approach to assessing language comprehension, focused solely on a child’s vocabulary, fails to accurately capture their language comprehension phenotype. Atypically developing children, for example, may acquire a large vocabulary without ever reaching the Syntactic Phenotype. Moreover, this vocabulary-centric approach may reduce the effectiveness of language therapy by emphasizing word learning over essential syntactic exercises needed for comprehensive language development.

A clinician-administered assessment of the three language comprehension phenotypes will enhance the characterization of language deficits, facilitate early diagnosis of language delays, and improve monitoring of syntactic language acquisition in children undergoing language therapy. To address this need, we developed the *Language Phenotype Assessment* (LPA), a 15-item tool specifically designed to evaluate language comprehension phenotypes in very young children. The LPA was administered to 116 neurotypically developing children aged 1.5 to 7 years. Results showed a linear increase in LPA scores from ages 1.5 to 5 (Figure 1), confirming its sensitivity to language comprehension development in early childhood. The progression of language comprehension phenotypes in typically developing children paralleled that of previously reported progression in autistic individuals [27]: 50% of neurotypical children attained the Command Phenotype by 1.6 years, the Modifier Phenotype by 3.0 years, and the Syntactic Phenotype by 3.7 years (Figure 2). All participants who acquired the Modifier Phenotype also acquired the Command Phenotype. Furthermore, all but one participant (97%) who acquired the Syntactic Phenotype also acquired the Modifier phenotype. The one remaining participant likely experienced lapses in attention while evaluating Modifier-level items.

Alongside the clinician-administered LPA, we invited parents to evaluate their children using the *Language Phenotype Evaluation Checklist* (LPEC). This 15-item LPEC mirrors the structure of the LPA, with three items assessing Command-level abilities, five items assessing Modifier-level abilities, and seven items assessing Syntactic-level abilities. LPEC scores also demonstrated a linear increase from ages 1.5 to 5 (Figure 3), supporting the effectiveness of the LPEC in tracking language comprehension development in young children. The progression of language comprehension phenotypes observed in typically developing children evaluated by parent-reported LPEC was the following: 50% attained the Command Phenotype by 1.6 years, the Modifier Phenotype by 2.7 years, and the Syntactic Phenotype by 3.0 years (Figure 5). The slight differences observed between the clinician- and parent-reported phenotype trajectories (Figure 2 and Figure 4) are likely due to item variation between the two assessments. Items in the LPA are not designed for parent reporting, nor are LPEC items suited for clinical evaluation, precluding the use of identical items across both tools.

Importantly, the developmental progression of language comprehension—advancing from the Command to the Modifier to the Syntactic Phenotype—is consistent across both clinician-assessments and parent-reports.

The strong correlation between LPA and LPEC scores (*r* = 0.78, *p* < 0.0001, Figure 4), along with substantial agreement demonstrated by Cohen’s weighted kappa = 0.73, 95% CI [0.60, 0.86], further supports the reliability of both tests in assessing a child’s language comprehension phenotype.

### 4.1. Acquisition of the Syntactic Phenotype by Participants Diagnosed with ASD According to the Clinician-Administered LPA

Based on the clinician-administered LPA, all neurotypical children aged 5 years and older achieved the Syntactic Phenotype. In contrast, only 39% of their peers with ASD reached this milestone (Figure S45 and Table S7). Among the remaining autistic participants aged 5 years and older, 37% attained only the Modifier Phenotype, 13% achieved the Command Phenotype, and 11% exhibited the pre-Command Phenotype.

All neurotypical children aged 4 years and older achieved the Modifier Phenotype. In contrast, only 66% of their peers with ASD reached this milestone (Table S8). Among the remaining autistic participants aged 4 years and older, 11% attained only the Command Phenotype, and 23% exhibited the pre-Command Phenotype.

Finally, all neurotypical children aged 3 years and older achieved the Command Phenotype. In contrast, only 74% of their peers with ASD reached this milestone (the remaining 26% exhibited the pre-Command Phenotype).

These observations suggest that the clinician-administered LPA may be a valuable tool for diagnosing and monitoring language comprehension deficits in individuals with ASD and other conditions frequently associated with language impairment.

### 4.2. Acquisition of the Syntactic Phenotype by Participants Diagnosed with ASD According to the Parent-Reported LPEC

A similar pattern was identified by the parent-reported LPEC. While all but one of the neurotypical children aged 5 years and older achieved the Syntactic Phenotype according to parent-reported LPEC, only 48% of their peers with ASD reached this milestone (Figure S46 and Table S7). Among the remaining autistic participants aged 5 years and older, 30% attained only the Modifier Phenotype, 9% achieved the Command Phenotype, and 13% exhibited the pre-Command Phenotype.

According to the parent-reported LPEC, all neurotypical children aged 4 years and older achieved the Modifier Phenotype. In contrast, only 68% of their peers with ASD reached this milestone (Table S8). Among the remaining autistic participants aged 4 years and older, 14% attained only the Command Phenotype, and 18% exhibited the pre-Command Phenotype.

According to the parent-reported LPEC, all neurotypical children aged 3 years and older achieved the Command Phenotype. In contrast, only 81% of their peers with ASD reached this milestone (the remaining 19% exhibited the pre-Command Phenotype).

### 4.3. Using LPA to Monitor Success of Language Therapy

The American Academy of Pediatrics (AAP) recommends universal screening of children aged 1.5 and 2 years for ASD, and also that individuals diagnosed with ASD begin to receive no less than 25 h per week of treatment within 60 days of identification [52]. However, language therapy delivery often faces three major challenges: (1) insufficient availability, (2) delayed intervention, and (3) misplaced focus.

First, many children receive too little therapy. Two-thirds of US children on the autism spectrum under the age of 8 fail to get even the AAP-recommended minimum treatment hours [53]. The problems range from the availability to general funding for early intervention programs [54,55,56]. Since the AAP’s 2007 recommendation of universal early screening, there has been a sharp increase in demand for ASD-related services [57]. However, according to a recent study, most states have reported an enormous shortage of ASD-trained personnel, including behavioral therapists (89%), speech-language pathologists (82%), and occupational therapists (79%) [57]. In many areas, children receive less than 5 h per week, falling far short of the recommended care levels [57].

Second, therapy often begins too late. Although ASD symptoms typically emerge in early development, the average age of diagnosis is around 4 years [58]. Current clinical guidelines [59,60] highlight diagnosis as a catalyst in the clinical pathway to initiate therapeutic intervention. Applying for an early intervention program and waiting for a therapist’s availability adds several additional months before therapy starts. As a result, most children usually do not begin therapy until 4.5 years of age. The scientific consensus, however, is that earlier interventions lead to a greater impact on developmental outcomes. In a recent study, Whitehouse et al. determined the efficacy of such preemptive intervention for ASD beginning during the prodromal period [22]. In the rater-blinded randomized clinical trial, the investigators compared preemptive intervention with usual care. Using community sampling, the investigators identified 104 one-year-old infants showing early behaviors associated with autism spectrum disorder. Each participant was randomized to receive either a preemptive intervention to go along with usual care or just usual care. The preemptive intervention regimen consisted of a 10-session social communication intervention. Each infant was assessed for a number of metrics at baseline (around age 1 year) and at age 3 years. The three-year-old reassessment included 89 participants, 45 of whom were in the preemptive intervention group. The intervention led to a reduction in ASD symptom severity, as well as reduced odds of ASD diagnosis. In the intervention group, 6.7% of participants were diagnosed with ASD compared to 20.5% in the usual care group. The number needed to treat to reduce the ASD diagnosis was 7.2 individuals. Other studies have also demonstrated that early intervention, particularly language exercises, significantly improves children’s outcomes [20,61,62,63,64] and is the greatest tool available to reduce the societal cost of treating ASD.

Third, language therapy for autistic children frequently emphasizes word learning and articulation, which are more straightforward goals. Vocabulary training is (1) quicker to implement, (2) highly valued by parents, (3) intuitive, since typically developing children amass extensive vocabularies before mastering syntactic language, and (4) reinforced by conventional language assessments, such as the Peabody Picture Vocabulary Test (PPVT-4) [31] and Expressive Vocabulary Test (EVT-2) [32]. However, this heavy emphasis on vocabulary often diverts attention from crucial syntactic language exercises. Critically, while word learning can occur across the lifespan, the acquisition of syntactic language is constrained by a well-defined critical period [6,16,65,66]. Widespread adoption of the LPA as a benchmark for language therapy success could shift the focus back toward syntactic-language training, allowing more children to reach their full linguistic potential.

### 4.4. Using LPA to Fine-Tune Language Therapy

For optimal language development, a structured educational sequence tailored to each child’s current abilities is essential, particularly in neurodiverse learners [67]. The LPA score can serve as a valuable guide for designing individualized language therapy curricula.

Children who have not yet attained the Command Phenotype (scoring below 2 on the LPA; see Table S6) are likely to benefit most from Command-level interventions. These may include learning the names of a few animal toys and practicing simple instructions, such as ‘*Take the cup/pencil/ball to the lion/dog/cat*’.

Those who have reached the Command Phenotype (scoring between 2 and 5) would be best served by focusing on Modifier-level language exercises. These activities involve learning colors, sizes, numbers, and following instructions, such as ‘*Select the small blue pencil*’ from a set of pencils, straws, and Lego pieces of different colors and sizes.

Children who have reached the Modifier Phenotype (scoring 6 and above) are ready to begin Syntactic-level language exercises. These may include practicing spatial prepositions (e.g., ‘*Put the lion on top/under/behind/in front of the giraffe*’) and working with syntactically-reversible sentences (e.g., ‘*Show the lion carries/rides the giraffe*’) (see a collection of exercises in [68]).

Critically, introducing Syntactic-level exercises to a child still at the Command Phenotype stage may be ineffective or even counterproductive, as it diverts time from more appropriate and accessible Modifier-level work. A finely tuned, stepwise approach guided by the LPA ensures that the intervention aligns with the child’s developmental readiness, thereby maximizing language acquisition progress.

### 4.5. Canonical Versus Noncanonical Word Order

Interpretation of any syntactic structure can be routinized. Consider the instruction, “put the green cup inside the blue cup”. This task can be completed through a simple algorithm: (1) lift the first-mentioned cup, and (2) place it into the second-mentioned cup. This type of stepwise response is an example of automatic, routinized action, similar to stopping at a red light. However, routinized responses do not equate to the acquisition of the Syntactic Phenotype, as they fail to generalize across the wide variety of sentence structures encountered in everyday language. In essence, reducing syntactic interpretation to an algorithmic routine does not translate to one’s ability to understand complex narratives, such as stories and fairy tales, where flexible and context-dependent comprehension is essential.

Naturally, in designing a test for language comprehension, we aimed to minimize opportunities for participants to rely on rote, algorithmic responses. Ideally, if we had prior knowledge of the specific tasks each individual had been trained on, we could have excluded those tasks. However, in a standardized test, it is impractical to avoid every task for which a participant might have a memorized solution. An alternative approach is to increase item complexity; the more intricate the items, the less likely participants are to have been explicitly trained on those sentence structures, reducing reliance on pre-learned algorithms. Yet, we also needed to avoid overly complex grammar that could overwhelm young children’s attention or working memory. Our chosen solution was to use noncanonical word order, creating a balance between discouraging rote responses and maintaining accessibility for all test-takers.

The results of this study corroborate our earlier findings that typically developing children aged 4 years and older comprehend canonical and noncanonical instructions equally well [29]. This study examined two sets of noncanonical instructions: stacking cups (item 10, Table 2) and spatial prepositions (item 12). Among neurotypical children who successfully followed the stacking cups instruction presented in canonical word order (item 9: “put the red cup inside the green cup”), 92% understood the instruction given in noncanonical word order (item 10: “inside the green cup, put the red cup”). Similarly, among neurotypical participants who followed the spatial prepositions instruction in canonical order (item 11: “put the giraffe under the monkey”), 88% understood the noncanonical version (item 12: “under the giraffe, put the elephant”). These findings indicate that, for the majority of typically developing children aged four and older, there is minimal difference in understanding between canonical and noncanonical word orders.

**Figure 6:**
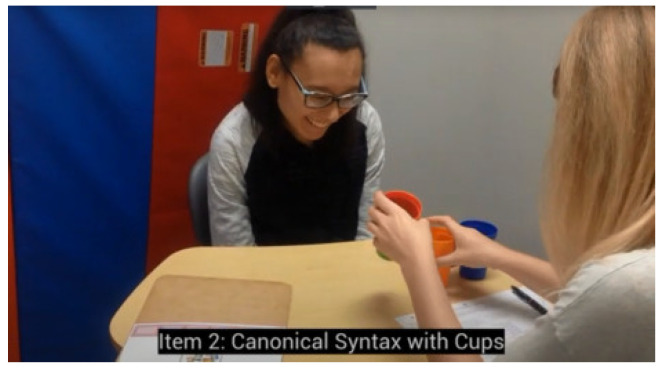
Reprinted with permission from [69]. Authors have obtained written parental consent to publish the video. Most autistic participants (18 to 21 years of age) were able to complete the canonical stacking cups task (e.g., “put the red cup inside the green cup”), but were unable to complete the same task under the condition of noncanonical word order (“inside the green cup, put the red cup”). Failing participants usually selected the correct cups but assembled them randomly. Most autistic participants have received over 15 years of intensive language therapy, and it is likely that their stacking cups routine has been automated through frequent training using canonical word order only. https://youtu.be/Hh7pkZB4ETU (accessed on 14 May 2025).

In contrast, among children aged 4 years and older diagnosed with ASD who successfully followed the canonical stacking cups instruction, only 76% comprehended the noncanonical version. Similarly, among those who successfully followed the canonical “spatial prepositions” instruction, only 60% comprehended the instruction given in noncanonical word order. These asymmetric results are similar to our previous research, which found that nearly half of autistic participants aged 18 to 21 years who successfully followed instructions in canonical word order failed the noncanonical version, suggesting a greater reliance on routinized responses (Figure 6).

### 4.6. The Modifier Phenotype

The acquisition of the Modifier Phenotype is sometimes mistakenly equated with learning the names of colors, sizes, and numbers. However, this reflects a fundamental misunderstanding. Learning an adjective, such as a color word, is mediated by Wernicke’s area, which associates a word with a corresponding color percept. This type of lexical association is confined to the posterior cortex and operates independently of the lateral prefrontal cortex (LPFC). As a result, individuals at the Command Phenotype stage are capable of forming such word-feature associations.

In contrast, assessing the Modifier Phenotype requires more than recognizing adjectives; it requires evaluating the ability to combine adjectives with nouns. For instance, a valid test might involve identifying “a long red pencil” placed among distractor objects varying in color, shape, and size—such as Lego pieces, drinking straws, and other pencils. Accordingly, all modifier-level items in the LPA (items 4 to 8, Table 2) were specifically designed to assess this combinatorial capacity.

The distinction between simply associating a word with a color and understanding phrases that integrate modifiers with nouns becomes especially apparent when human abilities are compared to those of non-human animals. Research has demonstrated that chimpanzees and some other animals are capable of learning words for different colors [70,71,72] and remember Arabic numbers up to nine [73,74,75,76]. However, no non-human animal has shown the ability to combine modifiers—such as color, size, and number—with nouns [77]. When faced with tasks that include distractors varying in color, number, and shape, such as items 4 to 8 in Table 2, animals consistently perform at chance levels, despite being familiar with individual words.

In short, the Modifier Phenotype—defined by the ability to integrate adjectives with nouns—is uniquely human. Remarkably, the median age at which typically developing children attain this distinctly human capability is 3.0 years.

## 5. Limitations

Learning words for colors and sizes alone does not equate to the acquisition of the Modifier Phenotype; however, without this foundational vocabulary, it becomes impossible to assess a child’s ability to integrate modifiers with nouns. Therefore, the LPA includes specific steps designed to demonstrate the meanings of individual words to a child prior to testing. Children who are unfamiliar with certain terms typically grasp their meanings quickly: each object used in the LPA is explicitly named, along with the relevant terms for colors, sizes, and numbers. Additionally, spatial prepositions are demonstrated and explained beforehand to ensure that the assessment focuses on sentence-level comprehension rather than vocabulary knowledge.

The use of manipulatives in the LPA poses a slight logistical challenge for clinicians, as picture-based tests are generally easier to store and transport. However, picture-based assessments can present additional difficulties for individuals with attentional issues. For instance, we previously described a case study of Peter, a 7-year-and-7-month-old fully verbal child with ADHD, whose performance on paper-based tests differed markedly from his performance with physical toys [29]. Peter received a standardized score of 74 on the Fluid Reasoning Index of the WPPSI-IV, placing him below 96% of the population, with scores of 6 on Matrix Reasoning and 5 on Picture Concepts. This IQ test required him to select the picture that best represented the correct answer. To further explore this discrepancy, we tested Peter with our proprietary paper-based assessment. He demonstrated an understanding of matrix analogies by successfully completing simpler items that involved “finding the same objects” and “integrating color, size, and number modifiers”. However, when asked to point to a picture representing “the man ate the whale” or “the whale ate the man”, Peter answered randomly. His performance on the paper-based test was below chance level, but his accuracy jumped to 100% when allowed to respond using physical objects. This suggests that the manipulatives were more engaging for Peter than paper-based options. A follow-up visit four months later, during which Peter had been taking 60 mg of Ritalin daily, yielded different results: he answered all paper-based items correctly. Since the goal of the LPA is to assess language comprehension rather than attention, we aimed to minimize the attentional component. Drawing from our experience with Peter and various studies indicating that manipulatives enhance attention more effectively than pictures [38,78,79], we developed the LPA to rely solely on manipulatives, completely eliminating pictures from the assessment.

While this study presents LPA norms in Figure 1, several limitations must be acknowledged. First, the normative data were derived from a relatively small sample of 116 children, which is insufficient for establishing robust, generalizable norms. Second, the sample was drawn predominantly from affluent neighborhoods, limiting the representativeness of the findings and potentially introducing socioeconomic bias. As a result, these norms may represent an idealized scenario and not accurately reflect the broader population. Future research should focus on developing comprehensive normative data based on larger and more diverse samples, including children from a range of cultural, linguistic, and socioeconomic backgrounds, to ensure the validity and applicability of the LPA across varied populations.

Another limitation of this study is its reliance on cross-sectional data, which restricts the ability to observe individual developmental trajectories. Future research should incorporate longitudinal designs to track language phenotype progression within individual children over time, offering deeper insight into the dynamics of language acquisition.

In addition to the clinician-administered LPA, this study explored the parent-reported LPEC. Although parents are often assumed to overestimate their children’s abilities due to factors such as social desirability bias [80], our findings reveal a strong correlation between clinician and parent scores (*r* = 0.78, *p* < 0.0001, Figure 4) and revealed substantial agreement between the assessments (Cohen’s weighted kappa = 0.73, 95% CI [0.60, 0.86]). This suggests that parents possess a nuanced and accurate understanding of their children’s language comprehension. Prior research has similarly shown that parent reports are generally consistent with clinician-administered assessments [81,82,83,84,85]. Together, these results support the validity of parent-reported assessments and highlight their potential as a complementary tool in language comprehension evaluation.

## 6. Conclusions

We present a 15-item Language Phenotype Assessment (LPA), a 10 min test designed specifically for evaluating syntactic language comprehension skills in children aged 1.5 to 5 years. The LPA items incorporate elements such as colors, sizes, numbers, spatial prepositions, and noncanonical syntax, posing a set of novel questions that participants have not encountered before. The total score for the LPA ranges from 0 to 15. The internal consistency of the LPA is high, with a Cronbach’s alpha of 0.92, indicating reliable measurement. Additionally, the assessment demonstrates excellent test-retest reliability and very high inter-observer agreement. Because the LPA does not depend on expressive language skills, it serves as a particularly valuable tool for assessing language comprehension development in minimally verbal children.

Using the LPA, we identified the typical ages at which children acquire the Command, Modifier, and Syntactic Phenotypes, thereby extending the foundational work of Piaget [51], Vygotsky [86], and Chomsky [87,88] by providing empirical detail on the specific developmental milestones of syntactic comprehension. Of the neurotypical children, 50% attained the Command Phenotype by 1.6 years of age, the Modifier Phenotype by 3.0 years of age, and the Syntactic Phenotype by 3.7 years of age. All neurotypical children acquired the Command Phenotype by 3, the Modifier Phenotype by 4, and the Syntactic Phenotype by 5 years of age.

Undetected delays in syntactic comprehension can have cascading effects on a child’s educational and social development. The LPA represents the first sentence-level language comprehension assessment specifically designed for very young children, spanning the developmental range of 1.5 to 5 years. Assessing children against the LPA benchmarks could become a part of a standard approach for early identification of language deficits. Earlier identification of comprehension challenges can facilitate the timely initiation of language interventions.

## Figures and Tables

**Table 1:** Comparison between language comprehension assessments for young children.

Language Assessment	Target Age Range (Years)	Comprehension Level Assessed (Word-Level vs. Sentence-Level)	Response Format (Pictures vs. Manipulatives)	Appropriate Syntactic Complexity for Ages 2–4	Assesses Language Comprehension Phenotypes
**Language Phenotype Assessment (LPA) [this article]**	1.5–5	Sentence-level	Manipulatives	Yes	Yes
**Peabody Picture Vocabulary Test (PPVT-4) [31]**	2.5–90+	Word-level	Pictures	No	No
**Expressive Vocabulary Test (EVT-2) [32]**	2.5–90+	Word-level	Pictures	No	No
**Preschool Language Scales (PLS-5) [33]**	0–8	Word-level for 0–4.5 years; sentence-level for 4.5–8 years	Manipulatives	No ^1^	No
**Token Test for children [34,35]**	3–14	Sentence-level	Manipulatives	No ^2^	No
**Clinical Evaluation of Language Fundamentals (CELF-5) [36]**	5–21	Sentence-level	Pictures	No	No
**Test for Reception Of Grammar (TROG) [37]**	4–18+	Sentence-level	Pictures	No	No

^1^ The PLS-5 uses overly complex sentences to assess sentence-level comprehension. For example, the easiest syntactic language comprehension task is Question 39: “Put the Mr. Bear in back of you,” “Put the Mr. Bear in front of me.” This task uses the combination of spatial prepositions (in back of, in front of) with pronouns (you, me) in the same sentence. The spatial prepositions and the pronouns present a significant challenge on their own. Combining the two into a single instruction makes the instruction significantly more difficult than each separately. Sensibly, the PLS-5 manual specifies that Q39 is targeted to 4-and-a-half-year-old children. The next syntactic language question after Q39 is Q46, which tests the understanding of nested sentences and is targeted at 6-year-old children. As a result, PLS-5 is not suited for evaluating language comprehension phenotypes in young children in the age range between 2 and 4 years. ^2^ The Token Test also suffers from overly complex instructions. The simplest Token Test spatial preposition task instructions are: “Put the red circle on the green square”, and “Put the green triangle on the red circle” [34,35]. The spatial prepositions and modifier domains both present a significant challenge to young children on their own. Combining them in a single sentence makes their comprehension even more daunting. The simplest possible spatial preposition domain instruction should only include a verb, a subject, an object, and a spatial preposition: e.g., “Put the lion on the giraffe.” The simplest possible modifier domain instruction should include a single modifier (size or color) and a noun. The longer-than-necessary instructions of the Token Test make it suboptimal for the assessment of language comprehension phenotypes in young language learners in the age range between 2 and 4 years.

**Table 2:** Clinician-observed *Language Phenotype Assessment* (LPA) items and associated tasks. When a child successfully completes at least three out of the four tasks, the item is scored as 1.

	Item	Tasks	
**Command items**	1. Give me an animal	1: Give me the giraffe 2: Give me the lion	3: Give me the elephant 4: Give me the monkey
	2. Give a cup/pencil to an animal	1: Give the cup to the monkey 2: Give the pencil to the giraffe	3: Give the cup to the elephant 4: Give the pencil to the lion
	3. Take an animal to another animal	1: Take the monkey to the giraffe 2: Take the giraffe to the lion	3: Take the elephant to the monkey 4: Take the lion to the elephant
**Modifier items**	4. Color integration	1: Give me a red straw 2: Give me a green pencil	3: Give me a yellow straw 4: Give me a blue pencil
	5. Size integration	1: Give me a big straw 2: Give me a small pencil	3: Give me a big pencil 4: Give me a small straw
	6. Color and size integration	1: Give me a big red straw 2: Give me a small green pencil	3: Give me a big yellow straw 4: Give me a small blue pencil
	7. Number integration	1: Give me two straws 2: Give me three pencils	3: Give me three straws 4: Give me two pencils
	8. Number and color integration	1: Give me three red straws 2: Give me two blue pencils	3: Give me three green straws 4: Give me two red pencils
**Syntactic items**	9. Stacking cups—canonical word order	1: Put the green cup inside the blue cup 2: Put the red cup inside the green cup	3: Put the green cup inside the yellow cup 4: Put the yellow cup inside the blue cup
	10. Stacking cups—noncanonical word order	1: Inside the blue cup, put the green cup 2: Inside the red cup, put the yellow cup	3: Inside the green cup, put the yellow cup 4: Inside the blue cup, put the red cup
	11. Spatial prepositions—canonical word order	1: Put the giraffe under the monkey 2: Put the elephant on the giraffe	3: Put the lion under the elephant 4: Put the monkey on the lion
	12. Spatial prepositions—noncanonical word order	1: Under the giraffe, put the elephant 2: On the monkey, place the elephant	3: Under the elephant, put the lion 4: On the lion, place the monkey
	13. Spatial prepositions behind, in front, between	1: Put the monkey next to you 2: Put the lion in front of you	3: Put the elephant behind you 4: Put the giraffe between you and I
	14. Mental reasoning with an object and a subject	1: If a boy washed a girl, who is clean? 2: If a tiger ate a lion, who has a full belly?	3: If a girl hit a boy, who is in pain? 4: If a boy fed a girl, who has a full belly?
	15. Mental reasoning with an object and a subject—passive voice	1: If a boy was showered by a girl, who is wet? 2: If a tiger was eaten by a lion, who has a full belly?	3: If a girl was pushed by a boy, who fell? 4: If a boy was fed by a girl, who has full belly?

**Table 3:** Parent-reported *Language Phenotype Evaluation Checklist* (LPEC) items. Answer choices were as follows: very true (2 points), somewhat true (1 point), and not true (0 points). A higher score indicates better language comprehension ability.

	Item
**Command items**	1. Knows names of common objects (cup, chair, car, pencil, etc.)
	2. Understands commands with pointing (e.g., if you point to the cup and say ‘bring me the cup’)
	3. Understands commands without pointing (e.g., ‘bring me the cup’)
**Modifier items**	4. Understands color modifiers (e.g., ‘green apple’ versus ‘red apple’ versus ‘green pencil’)
	5. Understands size modifiers (e.g., ‘big apple’ versus ‘small apple’ versus ‘big car’)
	6. Understands several modifiers in a sentence (e.g., ‘small green apple’)
	7. Understands size superlatives (can select the largest/smallest object out of a collection of objects)
	8. Understands NUMBERS (e.g., two apples vs. three apples)
**Syntactic items**	9. Understands possessive pronouns (e.g., your apple vs. her apple)
	10. Understands spatial prepositions (e.g., put the apple ON TOP of the box versus INSIDE the box vs. BEHIND the box)
	11. Understands verb tenses (e.g., I will eat an apple vs. I ate an apple)
	12. Understands the change in meaning when the order of words is changed (e.g., understands the difference between ‘a cat ate a mouse’ versus ‘a mouse ate a cat’)
	13. Understands simple stories that are read aloud
	14. Understands elaborate fairy tales that are read aloud (i.e., stories describing FANTASY creatures)
	15. Understands explanations about people, objects or situations beyond the immediate surroundings (e.g., “Mom is walking the dog,” “The snow has turned to water”)

## Data Availability

De-identified raw data from this manuscript are available from the corresponding author upon reasonable request.

## Supplementary Materials

The following supporting information can be downloaded at: https://www.mdpi.com/article/10.3390/children12060793/s1, The supplementary file provides additional data, figures, and methodological details that support the findings presented in the main article. It includes extended results, statistical analyses, and supplementary tables/figures referenced in the text, offering further insight into the study’s methodology and outcomes. References [89,90,91,92,93,94] are cited in Supplementary Materials.
